# Reducing prescribing of benzodiazepines in older adults: a comparison of four physician-focused interventions by a medical regulatory authority

**DOI:** 10.1186/s12875-021-01415-x

**Published:** 2021-04-08

**Authors:** Nigel Ashworth, Nicole Kain, Delaney Wiebe, Nancy Hernandez-Ceron, Ed Jess, Karen Mazurek

**Affiliations:** 1 Department of Medicine, University of Alberta, Edmonton, Canada; 2 Research and Evaluation Unit, College of Physicians and Surgeons of Alberta, 10020-100 Street NW, Edmonton, AB 2700T5J 0N3 Canada

**Keywords:** Benzodiazepines, Prescribing, Physicians, Interventions

## Abstract

**Background:**

The inappropriate and/or high prescribing of benzodiazepine and ‘Z’ drugs (BDZ +) is a major health concern. The purpose of this study was to determine whether physician or pharmacist led interventions or a simple letter or a personalized prescribing report from a medical regulatory authority (MRA) was the most effective intervention for reducing BDZ + prescribing by physicians to patients 65 years of age or older.

**Methods:**

This was a four-armed, one year, blinded, randomized, parallel-group, investigational trial in Alberta, Canada. Participants were fully licensed physicians (n = 272) who had prescribed 4 times the defined daily dose (4 + DDD) or more of any BDZ + to an older patient at least once in the 3^rd^ quarter of 2016. All physician-participants were sent a personalized prescribing profile by the MRA. They were then randomized into four groups that received either nothing more, an additional personal warning letter from the MRA, a personal phone call from an MRA pharmacist or a personal phone call from an MRA physician. The main outcomes were prescribing behavior change of physicians at one year in terms of: change in mean number of older patients receiving 4 + DDD BDZ + and mean dose BDZ + prescribed per physician. To adjust for multiple statistical testing, we used MANCOVA to test both main outcome measures simultaneously by group whilst controlling for any baseline differences.

**Results:**

All groups experienced a significant fall in the total number of older patients receiving 4 + DDD of BDZ + by about 50% (range 43–54%) per physician at one year, and a fall in the mean dose of BDZ + prescribed of about 13% (range 10–16%). However, there was no significant difference between each group.

**Conclusions:**

A personalized prescribing report alone sent from the MRA appears to be an effective intervention for reducing very high levels of BDZ + prescribing in older patients. Additional interventions by a pharmacist or physician did not result in additional benefit. The intervention needs to be tested further on a more general population of physicians, prescribing less extreme doses of BDZ + and that looks at more clinical and healthcare utilization outcomes.

**Supplementary Information:**

The online version contains supplementary material available at 10.1186/s12875-021-01415-x.

## Background

Much attention has appropriately been given to the current opioid epidemic [1]. Indeed, the province of Alberta has seen some of the highest rates of opioid usage in Canada, in a country that has one of the highest rates of usage in the World [2–4]. Less well-known is the use of benzodiazepines (BDZ) and related compounds that have also reached high levels [5–8]. Several studies have shown rates of BDZ usage in the over 65 yr population to be about 20%, with even higher rates in women, over 75yrs and in the institutionalized population [9–12].

Similar to opioids, there is evidence that significant proportions of BDZ prescribing is contributing to substance use disorder and are ‘diverted’ from legitimate sources [13, 14]. Kapil et al. 2014 found 7.7% of a sample of 1500 adults in the UK had misused BDZ at some time, and 55% of those had obtained the drug directly from a physician [15]. The number of prescriptions and the dosage prescribed by physicians is directly correlated with clinical evidence of substance use disorder (such as emergency visits or deaths due to overdose) [16, 17]. As many as half of the BDZ pills used by a highly selected population of patients with BDZ substance-use disorder were found to come from ‘legitimate’ prescriptions [13].

The combination of BDZ and other drugs in overdose is particularly severe, with one-third or more of fatal opioid overdose deaths having concomitant BDZ use [18–21]. Sun et al. 2017 calculated that eliminating concurrent use of BDZ and opioids could reduce hospital admissions and emergency room visits by as much as 15% [19]. BDZ may contribute to substance use disorder, particularly in older patients [7, 22–26]. Such patients (e.g. those aged 65 years and older) are especially susceptible to adverse reactions and events such as falls, fractures, and cognitive impairment, which appear to be most strongly correlated to the dose taken rather than the frequency or type of medication [27, 28]. In addition the prevalence of BDZ related substance-use disorder is high at over 10% and as high as 21% in 65 yr + patients admitted to a psychiatric inpatient unit [29, 30].

Attempts have been made to test different strategies for BDZ reduction, but these have shown only weak effects at best; and in a recent editorial Hayhoe and Lee-Davey 2018 state that “better evidence is urgently required for both drug and non-drug options [for BDZ withdrawal]” [31–33]. A non-systematic review of interventions for improving benzodiazepine prescribing concluded that “…many different interventions strategies are used Worldwide, with varying success” [34]. These BDZ reduction attempts have focused mainly on patient-related strategies such as interventions by family physicians, educational sessions, and psychosocial or pharmacological interventions [31, 32, 34–37]. Relatively few attempts have focused on interventions directed at reducing prescribing by physicians [38–40]. Lopez-Sepulveda et al. 2017 found a 35% reduction in potentially unsafe prescriptions of *Zolpidem* in volunteer clinics who received “training sessions, individualized feedback, clinical information, and financial incentives” [38]. Doctor et al. 2018 found a 9.7% reduction in opioid dosages prescribed for new patients up to 3 months after physicians were informed of one of their patients’ death by overdose, compared to a control group of physicians who were not given that knowledge [40]. Bachhuber et al. 2016 found widespread implementation of prescription monitoring programs in the United States have not led to any reduction in emergency room visits for BDZ overdose [39].

The College of Physicians & Surgeons of Alberta (CPSA) is the medical regulatory authority (MRA) for the province of Alberta, Canada. All drug dispenses from community pharmacies are entered electronically into a provincial drug repository, the Pharmaceutical Information Network (PIN). Data on the prescribing physician, the patient, the pharmacy, and medication dose, frequency, and total dose dispensed is uploaded within 48 h to the CPSA’s secure prescribing databases. In 2015 the CPSA began monitoring benzodiazepines and ‘z’ drugs (Additional file 1: Appendix) which provided a unique opportunity to both proactively select higher prescribing physicians, and to more rigorously investigate several different types of intervention(s) for potentially reducing benzodiazepine and ‘Z; drug (BDZ +) prescribing.

The objective of this study was to determine the most effective strategy for reducing BDZ + prescribing by physicians to older patients.

## Methods

This was a four-armed, one year, blinded, parallel-group, investigational trial. Ethical approval was obtained from the University of Alberta health research ethics board (Pro00065136) and informed consent was waived. Consent was waived by the ethics committee for several reasons: First, it was heavily in the public interest for the CPSA to intervene where public safety could be at risk. Second, the CPSA has the legal right and in fact the obligation to intervene as it sees fit, as delineated by law in the Health Professions Act of Alberta. Third, the ‘usual’ care would essentially be the same as group 4 which is the most onerous and stressful intervention, so in the trial those who take part actually have a good chance (75%) of receiving a “milder” intervention. Finally, a physician who refuses to take part in the research trial would automatically be given the most onerous intervention (group 4) which potentially could be deemed to be coercive in itself.

Physician-participants had full registration with the CPSA to practice in the province of Alberta, Canada who had prescribed greater than or equal to four times the defined daily dose (4 + DDD) of BDZ + to any patient aged 65yrs or older in the third quarter of 2016 (July 1 – September 30th, 2016). The DDD is the assumed average maintenance dose per day for a drug used for its main indication in adults [41].

Drug DDD values were obtained primarily from the WHO DDD/ATC Index [42]. The number of DDDs (i.e., the dose in multiples of the DDD) was used as the standard measure of dosing across all drugs and routes of administration within the benzodiazepines analytic class. The DDDs for a specific drug dispense were calculated as: Dispense DDDs = strength x quantity / drug DDD A patient’s total DDDs was calculated as: Patient DDDs = the sum of the DDDs for all drug dispenses to the patient in the time period analyzed /days in the time period analyzed.

To reduce the chance of a ‘regression to the mean’ effect the third quarter 2016 data was not used in the analysis. It was only used for the initial selection of presumably higher prescribing physicians.

We initially wanted to use ‘any BDZ + ’ prescription as the inclusion criteria given this might make more sense in this older patient population and lead to better generalization of any results. However, we had to use 4 + DDD ultimately for a number of reasons: 1) We realized that this should result in the flagging of approximately 300 physicians which is logistically all we could manage, using 3 + DDD would have resulted in over 800 physicians being flagged; 2) power calculations estimated that approximately 180 physicians would be required; and 3) 4 + DDD represents a high dose particularly in the older population, which would rarely, if ever, be justified clinically (Additional file 1: Appendix). Physicians who were actively engaged in any existing CPSA program (e.g. under active investigation because of a complaint) were excluded from the study.

The interventions occurred between the 4th quarter 2016 to 2^nd^ quarter 2017. Given that the CPSA has a legal responsibility to intervene in situations where patient safety may be threatened, all physicians in the trial were sent their own personal prescribing profile report encompassing the ‘control’ intervention [43]. This individualized report identifies patients to which the physician may have prescribed potentially harmful doses of opioids and/or BDZ + and indicates how the individual physician’s overall prescribing compares with a matched peer group.

Selected physician-participants were then randomized by computer-generated random selection (using Excel’s random number generator to generate a series of random numbers between 1 and 4) into the following four groups, with an equal chance that a physician-participants might be allocated to any group.

Group 1 ‘control’: no further intervention. This group is purely an ‘audit and feedback’ type intervention and had the lowest costs and resources.

Group 2 ‘letter’: An additional personalized letter from CPSA indicating the dangers of continuing to prescribe to specific patients and asking physician-participants to decrease dose if at all possible. There was also information on BDZ + prescription best practice and advice on how to reduce medications in patients receiving higher doses and links to external prescribing resources.

Group 3 ‘pharmacist’: Group 2 intervention plus physician-participants receives a phone call from the CPSA pharmacist who follows a semi structured conversation similar to the information in the letter for group 2. We chose a pharmacist group because these professionals are highly knowledgeable and respected as resources for drug information, the costs are cheaper than a physician and because they represent an interesting control for the ‘human’ contact in group 4.

Group 4 ‘physician’: Group 2 intervention plus physician-participants receives a phone call from one of two CPSA physicians who follows a semi structured conversation the same as in group 3. To date this has been the “usual” intervention used by the CPSA for higher prescribing physicians. This group also represents the most expensive intervention of the four groups.

## Outcomes

### Primary outcomes

Change in baseline (Q3 2015 – Q2 2016) to 1-year follow-up (Q4 2017 – Q3 2018) in:

1. Mean number of older patients (65 years or older) prescribed high doses (4 + DDDs) of BDZ + per quarter per physician (Mean_#)
2. Mean DDD of BDZ + prescribed to an older patient per quarter per physician (Mean_DDD).

### Secondary outcomes

1. Crude direct costs (to the MRA) of each intervention

We had no previous similar trials to help with sample size estimation. Hence, we calculated that we would need a sample size of ~ 45 physician-participants per group assuming 4 groups, using α = 0.05, and β = 0.8, and a moderate effect size of 0.25. To act conservatively we aimed to recruit approximately 50% more than we theoretically needed with ~ 70 physician-participants per group (total 280).

Allocation concealment was maintained by using non-research CPSA staff who matched the random generated group number to the list of flagged physicians, and who then coordinated the project and initiated the various interventions.

## Statistical methods

Baseline measures for the primary outcomes were calculated for each physician-participant by averaging the results for one year prior to the study selection i.e. 3^rd^ quarter 2015 through to end 2^nd^ quarter 2016. The interventions occurred between 4^th^ quarter 2016 and 2^nd^ quarter 2017. Then ‘one year’ follow up outcomes were calculated by averaging the results for one year after the study intervention i.e. 4^rd^ quarter 2017 through to end 3rd quarter 2018. Change in outcomes were calculated by subtracting the one year follow up values from the baseline. Differences were tested by ANOVA with Bonferroni post hoc testing where appropriate.

All direct costs were collected by CPSA staff for each intervention group. These included material (paper and postage), administrative (administrative time), managerial costs and professional fees (pharmacist and physician time). The cost of the prescribing monitoring system, other indirect or lost opportunity costs were not included.

To test changes in outcomes within groups a paired t-test and Wilcoxin signed-rank test were both performed.

To adjust for multiple statistical testing, we used MANCOVA to test both main outcome measures (change in ‘Mean_#’ and ‘Mean_DDD’ at one year) simultaneously by group whilst controlling for any baseline differences.

R statistics was used for the analyses (https://www.r-project.org/about.html).

## Results

**Fig. 1:**
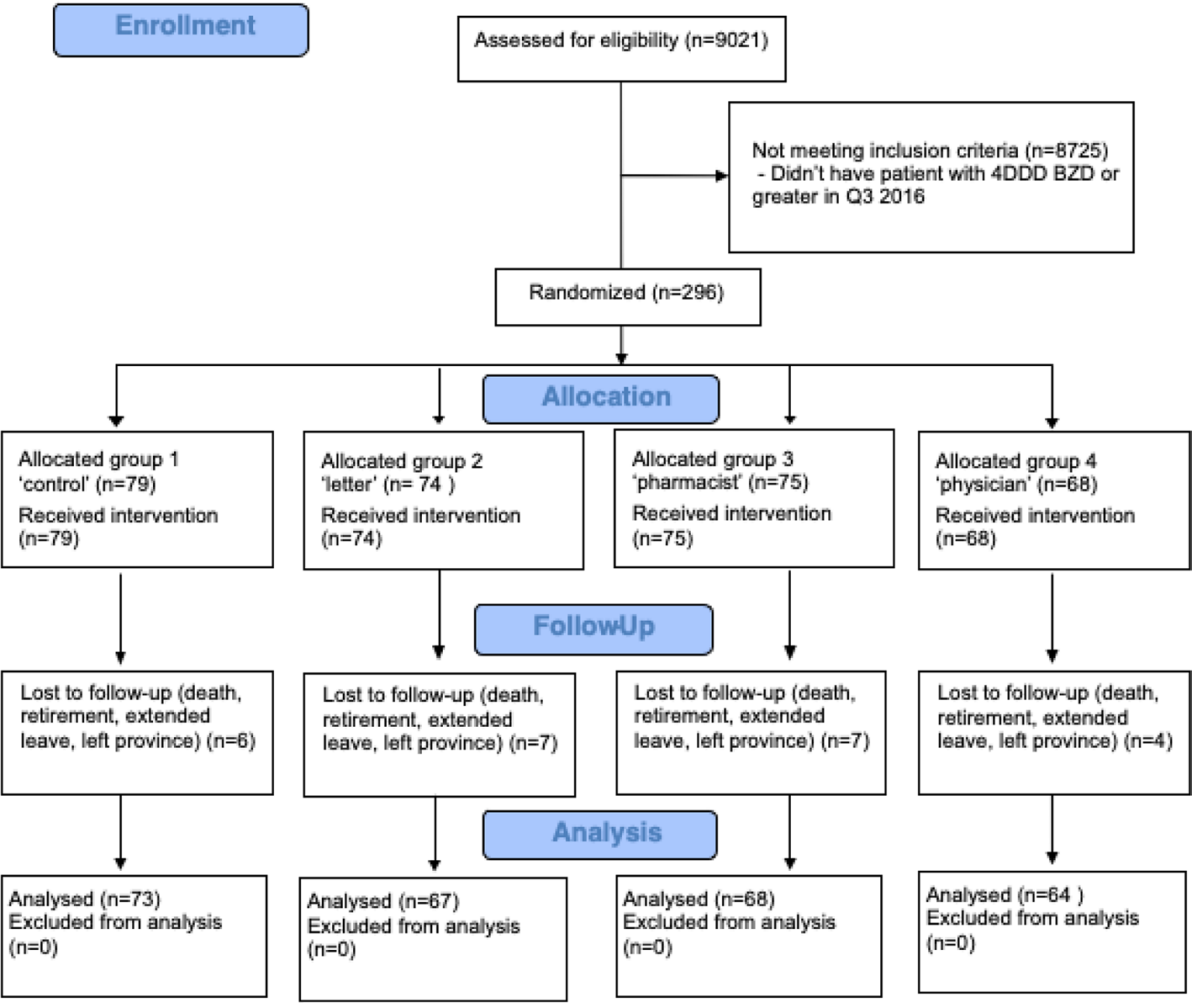
Consort flow diagram of trial

There were 9021 physicians with full registration in the province during the study period and 296 met the inclusion criteria and were entered into the trial. Twenty-four physicians (8.1%) did not complete the trial due to death, retirement, extended leave or relocation out of province. Hence, data was only analyzed on 272 physician-participants (Fig. 1).

Overall the included physician-participants were older than our mean age for the whole province and more heavily weighted towards male, and either family physicians or psychiatrists. Baseline data for the ‘control’ group had significantly more BDZ + patients and the ‘physician’ group had significantly lower baseline prescribed dosage than the other groups; otherwise there were no major differences between groups (Table 1). Five physician-participants were not registered for a full year prior to trial and so the baseline data was averaged for 6 months (2 physicians) and for 9 months (3 physicians).

**Table 1:** Baseline characteristics of physician-participants by intervention group (n = 272)

	Intervention Group				*P* value
	Control	Letter	Pharmacist	Physician	
Number of physicians (n)	73	67	68	64	
Age (mean/SD)	53.7 (10.6)	54.0 (12.0)	54.5 (11.4)	55.2 (12.2)	0.886
Male (%)	84	79	76	78	0.713
Family physicians (%)	85	81	85	92	0.301
Psychiatrists (%)	10	15	13	6	0.389
Baseline number of older patients prescribed high doses of BDZ + per quarter (mean/SD)	0.959 (1.001)	0.593 (0.696)	0.618 (0.656)	0.598 (0.727)	0.039
Baseline dose (DDD) of BDZ + prescribed to older patients per quarter (mean/SD)	1.065 (0.310)	1.089 (0.432)	1.074 (0.356)	0.935 (0.208)	0. 034

**Fig. 2:**
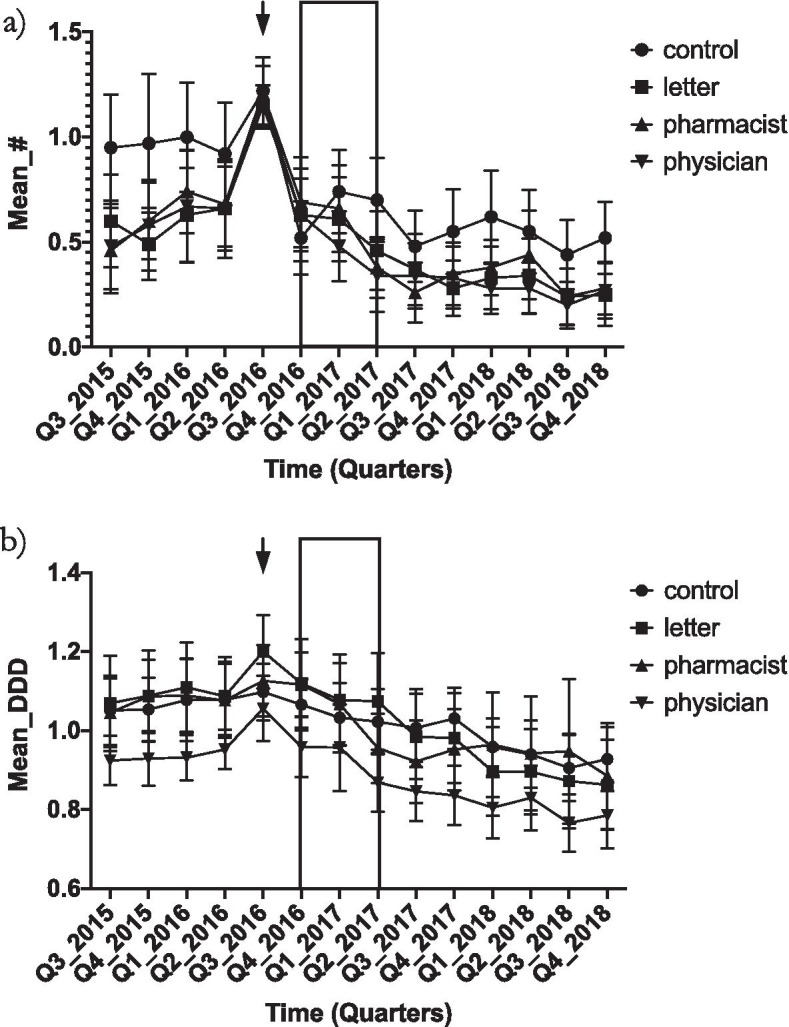
Mean number of older patients prescribed high doses of BDZ + per quarter per physician (**a**) and mean DDD of BDZ + prescribed to older patients per quarter per physician (**b**). Shaded area is the intervention period. Arrow is the quarter used for selection. Error bars represent 95% confidence interval

Prescribing rates for the 12 months prior to the start of the study were quite stable (Fig. 2). There is a clear decrease in both primary outcomes that appears to begin towards the end of the intervention (Q2 2017) and continues till the end of the official follow up period (Q3 2018) (Fig. 2).

The change from baseline to 1-year follow up was statistically significant for all groups but between-group comparisons were not significant for any outcome (Table 2). The numbers of older patients receiving high doses of BDZ + dropped by about 50% (range 43–54%) and the total dosage given to all older patients fell by about 13% (range 10–16%) (Table 2).

**Table 2:** Decrease in the number of older patients prescribed high doses of BDZ + per quarter per physician and decrease in BDZ + prescribed (DDD) to older patients per quarter per physician (Mean ± standard error) over one year

	Control	Letter	Pharmacist	Physician
Decrease in number patients	0.42 ± 0.1 (44%) **	0.30 ± 0.1 (50%)**	0.27 ± 0.2 (43%)*	0.32 ± 0.2 (54%)**
Decrease in BDZ + prescribed	0.11 ± 0.10 (10%)*	0.18 ± 0.1 (16%)**	0.12 ± 0.1 (11%)*	0.13 ± 0.05 (13%)**

*DDD* Defined daily dose

^*^ statistically significant using paired t-test and Wilcoxin signed-rank test at < 0.05 level for change within groups, between group differences were not significant using MANCOVA

^**^ statistically significant using paired t-test and Wilcoxin signed-rank test at < 0.001 level for change within groups, between group differences were not significant using MANCOVA

Costs for each intervention ranged from a low of CAN$12.76 per physician-participants in the ‘control’ group to a high of CAN$81.71 per physician-participants in the ‘physician’ group (Table 3).

**Table 3:** Direct costs (CAN$) for each intervention

	Intervention Group			
	Control (n = 73)	Letter (n = 67)	Pharmacist (n = 68)	Physician (n = 64)
Professional Fees^a^	0.00	0.00	720.00	3,000.00
Shipping	148.00	134.00	136.00	126.00
Envelopes	18.50	16.75	17.00	15.75
Paper	14.80	6.70	6.80	6.30
Administrative	750.00	750.00	1,000.00	2,000.00
Total	931.30	907.45	1,879.80	5,148.05
Cost/participant	$12.76	$13.96	$27.64	$81.71

^a^Professional fees were based on hourly rates

## Discussion

All four interventions appeared to produce similar improvements in terms of numbers of older patients prescribed high doses of BDZ + with a drop of 50% at one year and also similar drops in total dosage of BDZ + prescribed to older patients of about 10%. No one group was superior to another, however.

We deliberately chose what we would consider an extreme sample of patients who were receiving a very high dose of BDZ + for their age. Therefore, the overall numbers per quarter are quite low at about 1 patient per physician. In addition, we should emphasize that these physicians are clearly on the ‘extreme’ side of the prescribing curve and therefore may well not be representative of the general physician population.

There is a large direct cost savings, of about 85% (see Table 3), to MRAs by switching from using a physician (which is typically the standard approach currently) to just sending a prescribing report. The ultimate savings would depend on whether this intervention would work equally well in a different more generalizable patient and physician population.

Ivers et al. 2012 in their Cochrane review demonstrated that audit-and-feedback interventions in general produce very small changes in the order of approximately 4% [44]. At present, there are no other investigations similar to this in the literature, allowing for the conclusion that the increased attention from a medical regulatory authority might have a greater effect on physician behavior than from other sources. One should be cautious however given our study was clearly of ‘extreme outliers’ and so one might expect much greater shifts in behavior than the Cochrane review found in a more general population.

There was no ‘pure’ control group (who received no intervention at all) in this study because it would have been difficult to justify legally and ethically. This means that there remains uncertainty regarding whether there was an actual effect of the prescribing profile report sent to all physicians or whether there was a coincidental general decrease in prescribing due to some unknown environmental effect. For example, it is possible that there was a governmental intervention with legislation change or perhaps a targeted media campaign that coincided with the start of the trial; however, the research team is unaware of any potential influencers that may have occurred. In addition, prescribing was clearly stable in these physicians the one year prior to the trial, and then fell at the onset of the trial for the next year at least. It is also possible that the trial was underpowered to pick up smaller differences between interventions (i.e. type II error) although ultimately, we enrolled almost 50% more physician-participants than we originally intended based on our original power calculations. It is also conceivable that the interventions from physicians and pharmacists might have a longer lasting effect than the letter. The changes at one year continue to be maintained for all interventions.

Other limitations of the study include the lack of certainty that the reductions in BDZ + prescribing are actually benefiting patients or resulted in physicians denying BDZ + s to patients that clinically require them. We were unable to link this data to any databases that contained clinical outcomes unfortunately because of time and resource limitations. Additionally, it is possible that physicians in the trial simply transferred these high dose BDZ + patients to other physicians who were not in the trial. Furthermore, the reduction in BDZ + dose may have caused the physician to raise the dose of or introduce a new drug, equally dangerous, to compensate, such as an antidepressant. Patients may have simply left the physician’s practice and obtained their BDZ + s elsewhere, or patients might have died or been hospitalized. However, given the allocation was randomized then there is no reason to suspect that any of these issues would have systematically varied between groups. This study measured BDZ + prescriptions dispensed in community pharmacies in Alberta and whether the patient actually consumed the prescribed dose, diverted it to a third party or disposed it remains uncertain. Finally, as we mentioned previously selection bias from the inclusion criteria, may limit the generalizability of these results to other patient groups or to other prescribers of BDZ + .

## Conclusions

A personalized prescribing report alone sent from the MRA appears to be an effective intervention for reducing very high levels of BDZ + prescribing in older patients. Additional interventions by a pharmacist or physician did not result in additional benefit. The intervention needs to be tested further on a more general population of physicians, prescribing less extreme doses of BDZ + and that looks at more clinical and healthcare utilization outcomes.

## Supplementary Information

**Additional file 1.**

## Data Availability

Non-identifiable original data may be available on request to corresponding author. We obtained ethical approval for the trial (University of Alberta, Alberta health services, and Covenant health joint health research ethics board, Pro00065136) and applied for and received exemption from obtaining informed consent. This was granted because: the CPSA has the legal authority/obligation under the health professions act of Alberta (Health Professions Act, R.S.A. 2000, c H-7) to intervene regardless, the most onerous intervention arm of the trial (group 4) was our ‘usual care’ and hence recruiting participants could easily be perceived as coercive (“if you don’t agree to participate you will be allocated to the severest group anyway”), physicians are not seen as disadvantaged and because it was of critical importance for the public good. Also, two arms of the trial investigated the effect of contact with the medical regulator which we couldn’t do if the physicians were aware.

## Abbreviations

BDZ+: Benzodiazepines and ‘Z’ drugs
Z drugs: Imidazopyridines (e.g. zolpidem), Cyclopyrrolones (e.g. zopiclone) and pyrazolopyridines (e.g. zaleplon)
Older patients: Patients 65 years or older in Q3 2016
DDD: Defined daily dose (The assumed average maintenance dose per day for a drug used for its main indication in adults)
4+DDD: Four or more times the defined daily dose
MRA: Medical regulatory authority
CPSA: The College of Physicians & Surgeons of Alberta
Mean_#: Mean number of older patients (65 years or older) prescribed high doses (4+DDDs) of BDZ+ per quarter
Mean_DDD: Mean DDD of BDZ+ prescribed to older patients (65 years or older) per quarter
