# Supplementary material for: Reducing prescribing of benzodiazepines in older adults: a comparison of four physician-focused interventions by a medical regulatory authority
Source: BMC Fam Pract. 2021 Apr 8;22:68. doi: 10.1186/s12875-021-01415-x (PMC8034172; doi:10.1186/s12875-021-01415-x)
Supplement: Supplementary file 1 — Additional file 1. [file 12875_2021_1415_MOESM1_ESM.docx]

Appendix: Benzodiazepine and Benzodiazepine-like drugs (Z-Drugs) available in Alberta with typical maximum and daily defined doses in <65yrs, and usual indications for selected benzodiazepines, Canada 2016. http://www.cpsa.ca/tpp/tpp-medication-list/

| Drug | Maximum indicated dose (mg)^1^ | Daily defined dose (mg)^2^ | 4 x Daily defined dose (mg) | Health Canada Indication |
| --- | --- | --- | --- | --- |
| **Long-acting** | | | | |
| Chlordiazepoxide | 40 | 30 | 120 | Anxiety disorders |
| Clorazepate | 60 | 20 | 80 | Anxiety, panic, seizure disorders, and alcohol withdrawal |
| Diazepam | 40 | 10 | 40 | Anxiety disorders, perioperative medication, seizure, skeletal muscle spasticity, and alcohol withdrawal |
| Flurazepam | 30 | 30 | 120 | Insomnia |
| **Intermediate-acting** | | | | |
| Alprazolam | 10 | 1 | 4 | Anxiety and panic disorder |
| Bromazepam | 60 | 10 | 40 | Anxiety disorders |
| Clobazam | 80 | 20 | 80 | Seizure disorders |
| Clonazepam | 20 | 8 | 32 | Seizure disorders |
| Lorazepam | 4 | 2.5 | 10 | Perioperative medication, anxiety and seizure disorders |
| Nitrazepam | 10 | 5 | 20 | Insomnia, seizure disorders |
| Oxazepam | 120 | 50 | 200 | Anxiety disorders, alcohol withdrawal |
| Temazepam | 30 | 20 | 80 | Insomnia |
| **Short-acting** | | | | |
| Triazolam | 0.5 | 0.25 | 1 | Insomnia |
| **‘Z’ drugs** |  |  |  |  |
| Zaleplon | 20 | 10 | 40 | Insomnia |
| Zolpidem tartrate | 10 | 10 | 40 | Insomnia |
| Zopiclone | 7.5 | 7.5 | 30 | Insomnia |
